# Evaluating the learning curve in robot-assisted laparoscopic total hysterectomy: single-port versus multi-port Da Vinci platforms

**DOI:** 10.1007/s11701-025-02928-8

**Published:** 2025-11-01

**Authors:** Riccardo Vizza, Simone Garzon, Giacomo Corrado, Valentina Bruno, Ermelinda Baiocco, Andrea Giannini, Stefano Uccella, Enrico Vizza

**Affiliations:** 1 Unit of Obstetrics and Gynecology, Department of Surgery, Dentistry, Pediatrics, and Gynecology, AOUI Verona, University of Verona, Piazzale A. Stefani 1, Verona, 37126 Italy; 2 Dipartimento di Scienze per la salute della Donna e del Bambino e di Sanità Pubblica, UOC Ginecologia Oncologica, Fondazione Policlinico Universitario A. Gemelli, IRCCS, Rome, Italy; 3 Gynecologic Oncologic Unit, Department of Experimental Clinical Oncology, IRCCS Regina Elena National Cancer Institute, Rome, Italy; 4 Unit of Gynecology, Department of Medical Surgical Sciences and Translational Medicine, Sant’Andrea Hospital, Sapienza University, Rome, Italy

**Keywords:** Learning curve; Robot-Assisted hysterectomy, Da Vinci Single-Port, Da Vinci Multi-Port

## Abstract

The aim of this study is to assess and compare the learning curves of the Da Vinci S Multi-port (MP) and Da Vinci Single-Port (SP) platforms for total hysterectomy, with particular attention to how prior MP experience influences SP performance. This is a retrospective comparative study on patients undergoing robot-assisted total hysterectomy (benign or early endometrial cancer indication) with the MP and SP systems. All operations were performed by the same surgeon, and cases performed with the SP system were performed after the MP system. Operating time was used as the main indicator of the learning curve, analyzed by the CUSUM method. Data were processed with R software, and differences between groups were evaluated with t-test and Chi-squared test (significance *p* < 0.05). A total of 147 patients were analyzed (73 MP, 74 SP). Mean operating times were similar between the two groups (117 min MP vs. 114 min SP). Hemoglobin drop was significantly lower in the SP group (− 1.18 g/dL vs. − 2.07 g/dL, *p* < 0.05). CUSUM analysis showed that the learning curve of the MP system reaches the mastery phase after 50 cases, while 13 cases are sufficient for the SP system when initiated after having mastered the MP system. The learning rate was − 0.3 min/case for MP and − 0.009 min/case for SP. In conclusion much of the experience gained with the Da Vinci Multiport System is transferable to the SP platform, allowing for a shorter learning curve and rapid achievement of surgical mastery with similar learning phases.

**Supplementary Information:**

The online version contains supplementary material available at 10.1007/s11701-025-02928-8.

## Introduction

The Da Vinci SP© is a specialized variant of the Da Vinci surgical system tailored for single-port surgery. It utilizes a single robotic arm that passes through one small skin incision. This is made possible by its design, which includes a single flexible camera and three multi-articulated robotic instruments. All these instruments employ two points of articulation within the body, allowing for better angulation toward the surgical field.

The Da Vinci Single Port by Intuitive Surgical, Inc. (Sunnyvale, CA, USA) received approval for gynecologic surgery in South Korea in 2019 and in Japan in 2022. In the United States, while SP was approved in 2019 for select otolaryngology and urology procedures, the approval for gynecologic surgery is still pending. In the EU, the Da Vinci SP has been approved for gynecological procedures in 2024.

Since 2019, the SP system has been demonstrated feasible for several major robot-assisted operations. SP surgery technique was introduced into clinical practice to perform cholecystectomy, colorectal surgery, urological and gynecological surgery, with encouraging preliminary results [1–4].

Recent comparative studies in gynecology have evaluated SP versus Xi for benign hysterectomy and endometrial cancer staging, reporting broadly comparable safety with platform-specific differences in operative time, docking, blood loss, and recovery, thereby contextualizing SP adoption in our field [4–7].

Given that the SP system was introduced after widespread adoption of the MP platform, it is crucial to understand whether the competencies gained with MP robot-assisted surgery are transferable to SP procedures. This has important implications for surgical training, patient safety, and the efficient integration of new technology into clinical practice. In this context, analyzing the learning curve (LC) becomes particularly relevant, as it represents the relationship between a surgeon’s experience and patient outcomes, and it is crucial to estimate the number of patients at risk of suboptimal outcomes during the learning process [8]. Our study aims to define the learning curve for the SP platform and the MP platform, with particular attention to how prior MP experience influences SP performance.

## Methods

We conducted a retrospective study to compare the learning curve for the procedure of total hysterectomy performed using the Da Vinci SP© (Intuitive Surgical, Inc., Sunnyvale, CA, USA) system versus the Da Vinci S Multi-port© (Intuitive Surgical, Inc., Sunnyvale, CA, USA) system. The study was approved by the Ethics Committee of the Regina Elena National Cancer Institute of Rome, Italy (RS: 322/IRE/25). Informed consent to surgical intervention, including consent for the retrospective use of clinical data, was obtained from all the patients in accordance with local and international legislation (Declaration of Helsinki) [9].

We retrospectively reviewed the prospectively maintained operative room database and identified all consecutive total hysterectomy procedures performed with robotic platform by the same single surgeon (EV). We selected the first cases performed by the surgeon with MP and SP system. The involved surgeon had already extensive background in laparoscopic surgery and advanced gynecological oncological procedures before the beginning of the study. The multi-port (MP) cohort (July 2010–September 2011) was deliberately selected to capture the surgeon’s initial robotic learning period, at a time when he had no prior experience with robotic surgery. The single-port (SP) cohort (June 2024–April 2025) reflects subsequent platform adoption after mastery of MP. Accordingly, the present analysis is designed to characterize a within-surgeon platform transition rather than a contemporaneous, head-to-head platform comparison.

We identified and included all consecutive robotic-assisted type A and type B1 hysterectomies [10] performed for benign gynecological condition or clinical stage I endometrial cancer. Variables of interest, such as patient demographics, surgical indications, operative times, and estimated blood loss, were retrieved from a prospectively maintained institutional database or collected from medical records by trained physicians.

For the MP group, the da Vinci S system with three robotic arms was used. After establishing pneumoperitoneum, three 8-mm da Vinci robotic trocars were inserted, together with a 10-mm assistant trocar.

For the SP group, a single 2.5-cm incision was made at the lower rim of the umbilicus and carried down to the fascia, which was then opened along the body’s longitudinal axis. The leading edge of the folded port was introduced into the incision with a downward motion while countertraction was applied with retractors. A small Intuitive access port was placed, and pneumoperitoneum was established by insufflating to 12 mm Hg. In SP cases, suction was obtained with a flexible suction tube passed alongside the 2.5-mm single-port cannula and directly controlled by the console surgeon. In MP cases, suction and irrigation were provided by the bedside assistant through the assistant trocar. Docking is faster with SP owing to single-incision access and the lack of multi-arm spacing/alignment [4]. These differences did not alter patient selection, the hysterectomy steps, or the learning-curve analysis framework.

### Statistical analysis

Patient demographics and perioperative variables were summarized using standard descriptive statistics, as appropriate, overall and stratified based on the surgical platform: the single-port group (SP) and the Da Vinci S multi-port group (MP). Comparisons between the two groups were performed for continuous variables using the Student’s t-test, while differences in nominal categorical variables were assessed with the Chi-squared test.

For visualization of the learning trend in raw operative times, we report the learing rate in min/case and R2 = 1 − SSE/SSTR (SSE = Sum of Squared Errors, SSTR = Total Sum of Squares), which represents the proportion of variance in OT explained by case order. R2 was used only to summarize the linear trend in OT and was not used for CUSUM-based mastery determination.

The cumulative sum (CUSUM) methodology was used for the learning curve analysis, and the primary endpoint was the operative time as best surrogate of learning and proficiency in using the robotic-surgical platform. All surgical cases were sorted by surgical dates. Data for each patient in the series were plotted on a chart from left to right on the x-axis, while the y-axis represents the CUSUM value of operative time. The CUSUM of the operative time was defined as follows: *S*_*0*_*​=0; S*_*i*_ *= S*_*i−1*_ *+ (OT*_*i*_ *−* $\:\overline{OT}$*).* Where *S*_*i*​_ is the CUSUM value for case *i*, S_i−1_ is the CUSUM value for the previous case. *OT*_*i*_​ is the observed operative time for case *i*, $\:\overline{OT}$ is the mean operative time defined as the cohort-level mean operative time computed across the entire series for that platform (MP and SP calculated separately and held constant within each series). The initial CUSUM value is 0 so *S*_*0*_*​=0*. Additionally, for both groups we plotted the raw operative times in chronological order, with a trend line representing the learning rate, modeled through linear regression. We defined the onset of the mastery phase as the first global maximum of the CUSUM curve (slope change from positive to negative), followed by a sustained decrease of the curve, consistent with most subsequent operative times falling below the cohort mean.

Statistical significance was considered with a p-value < 0.05. All statistical analyses were performed with R Statistical Software (v4.1.2; R Core Team 2024).

## Results

### Demographic and clinical characteristics

A total of 73 consecutive patients who underwent Da Vinci S multi-port (MP) hysterectomy between July 2010 and September 2011, and 74 consecutive patients who underwent Da Vinci SP single-port (SP) hysterectomy between June 2024 and April 2025 were identified and included in the analysis. Demographic and clinical characteristics are summarized in Table 1.

The two groups did not differ in terms of surgical indications. In the MP group, 56 patients had endometrial cancer and 17 had benign gynecological conditions (including endometrial hyperplasia and uterine fibromatosis). In the SP group, 63 patients had endometrial cancer and 11 had benign conditions. The two groups differed significantly in terms of age (mean 58 years in MP vs. 63 years in SP, *p* < 0.05), while they were comparable in terms of body mass index (mean BMI 31 kg/m² in MP vs. 29 kg/m² in SP). Regarding operative time no statistically significant difference was observed (*p* > 0.05), with a mean duration of 117 min in the MP group and 114 min in the SP group. In contrast, the mean postoperative hemoglobin drop was significantly lower in the SP group (1.18 g/dL) compared to the MP group (2.07 g/dL), with this difference reaching statistical significance (*p* < 0.05).

**Table 1:** Demographic and clinical characteristics

Characteristics	Da Vinci MP group (*n* = 73)	Da Vinci SP group (*n* = 74)	*p*
Mean Age (years)	58 (SD: 10.9)	63 (SD: 12.2)	0.011
Mean BMI (kg/m²)	31 (SD: 8.1)	29 (SD: 7.3)	0.055
Diagnosis	EC *n* = 56, Benign cases *n* = 17	EC *n* = 63, Benign cases *n* = 11	0.193
Mean operative Time (minutes)	117 (SD: 42.7)	114 (SD: 34.2)	0.658
Mean Hb drop (g/dL)	2.07 (SD: 1.1)	1.18 (SD: 0.91)	0.0000327

### Learning curve analysis

All procedures were performed by the same surgeon; the initial multi-port robot-assisted procedures were performed by the surgeon at a time when he had no prior experience in robot-assisted surgery. In contrast, the first SP cases were carried out after the surgeon had already gained substantial experience with multi-port robot-assisted surgery, although he had no prior clinical experience specifically with the SP platform.

**Fig. 1:**
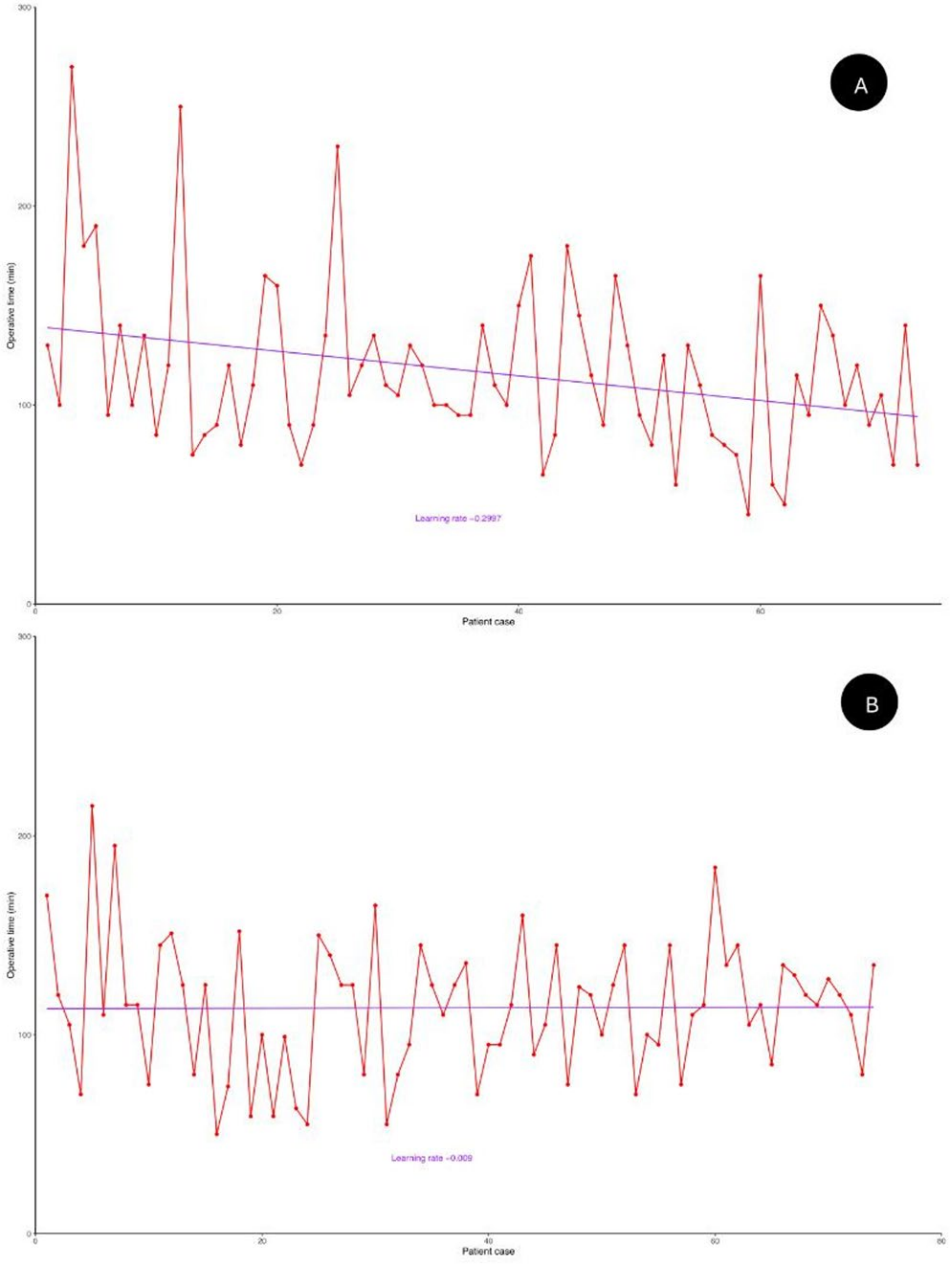
(**A**) Operating time as a function of the number of interventions in MP group (**B**) Operating time as a function of the number of interventions in SP group

**Fig. 2:**
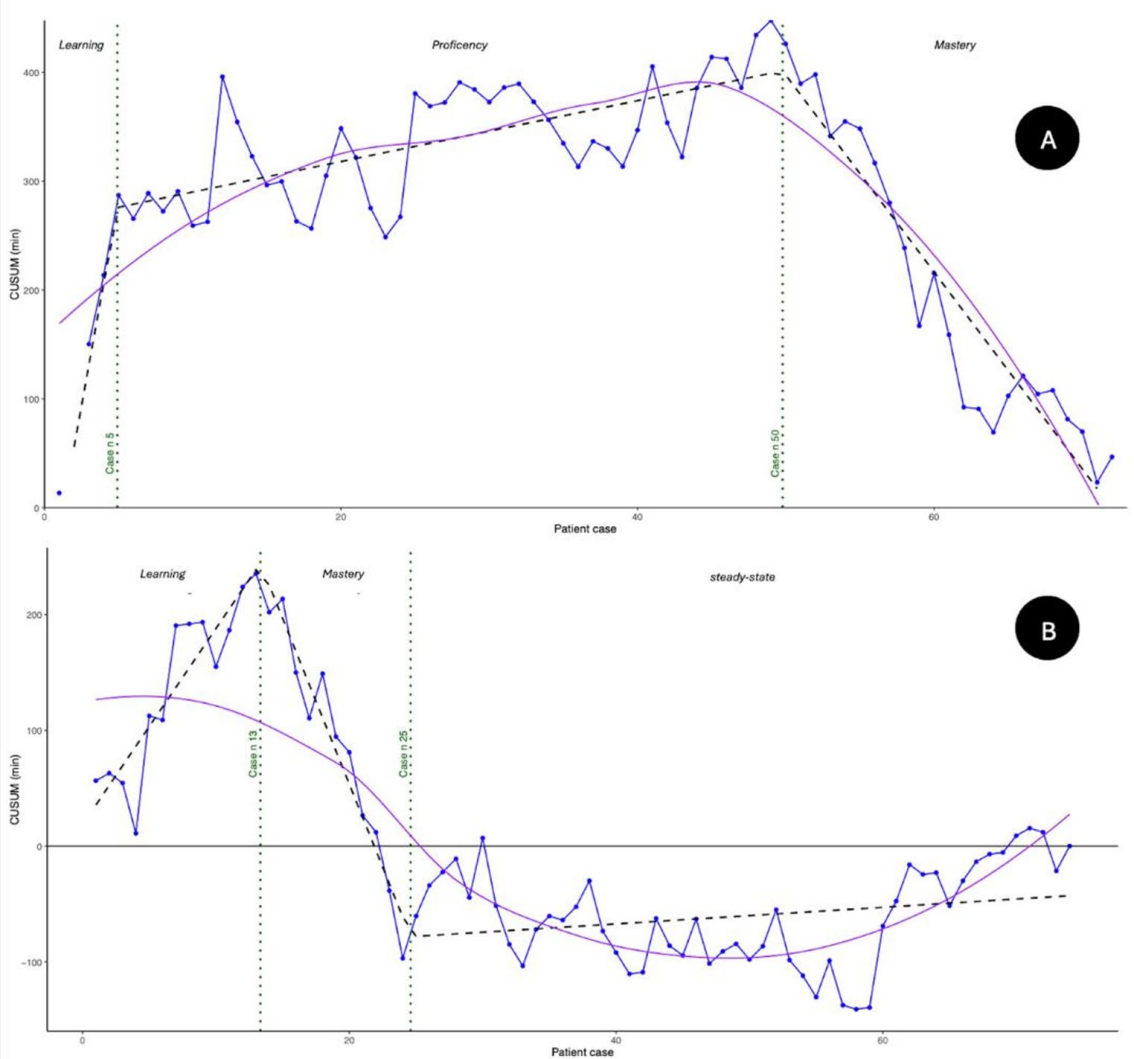
(**A**) CUSUM Analysis of Operative Time for MP group (**B**) CUSUM Analysis of Operative Time for SP group

A marked reduction in operative time was observed with increasing experience in the MP group, showing a learning rate of approximately − 0.3 min per case, indicating a steeper learning curve (Fig. 1A). Conversely, the SP group demonstrates a much more gradual decline in operative time, with a learning rate of − 0.009 min per case, reflecting a nearly flat learning curve (Fig. 1B). The CUSUM analysis of operative times in the MP group (Fig. 2A) identifies three distinct phases: A learning phase, characterized by a steep upward slope of the curve, corresponding to longer-than-average operative times. A proficiency phase, where the curve continues to rise but with a reduced slope. A mastery phase, marked by a downward slope, indicating operative times below the average.

In the MP group, the transition to the mastery phase occurred after 50 cases. In contrast, the CUSUM curve of the SP group (Fig. 2B) reveals a different pattern. The learning phase is shorter, with a rapid transition directly into the mastery phase without a clearly distinguishable proficiency phase. This mastery phase, marked by a consistent downward trend in the CUSUM curve, begins around case 13 and continues until case 25. Beyond this point, the curve flattens, indicating stabilization of operative performance and times oscillating closely around the mean. R^2^ for MP and SP CUSUM curves is respectively 0.897 and 0.862.

## Discussion

The comparative analysis of the learning curves showed that, although there was no statistically significant difference in mean operative times between the da Vinci S Multi-port and the da Vinci Single-Port platforms, the progression of surgical proficiency differed markedly. Specifically, the two platforms exhibited distinct learning curve patterns, indicating differences in the rate and trajectory of skill acquisition.

The CUSUM learning curves showed that the SP system reached the mastery phase after 13 procedures, whereas the multi-port MP system required 50 (Fig. 2B vs. Fig. 2A). This compressed trajectory is consistent with the principle of positive skill transfer: once a surgeon has internalized the psychomotor and cognitive workflows of console-based robot-assisted surgery, additional platforms that share similar hand-controller geometry, instrument kinematics and visual feedback impose a far smaller cognitive load. Consequently, only the platform-specific nuances (e.g. adaptation of operative angle and working distance, preventive hemostasis due to smaller instruments) must be learned, while core skills, camera control, three-dimensional depth perception, management of the controls and wristed articulation are already automatized. Comparable accelerations have been reported when surgeons move from MP to SP systems in radical prostatectomy [11, 12].

The only study in the literature reporting a CUSUM analysis for hysterectomy using the Da Vinci SP system is the study by Higuchi et al. (2025) [6], which identified a proficiency phase after the eighth case and a mastery phase after case number 29. In that study, all procedures were performed by seven gynecologists with extensive MP experience.

The MP curve displays the canonical three phases described in surgical education theory. In the initial learning phase, the curve has a steep upward slope, indicating that the operator is taking longer than the mean operative time. As declarative knowledge is converted into automatized motor patterns, the curve enters the proficiency phase; where the curve begins to flatten but still has an upward slope. Finally, the mastery phase is reached when the curve reverses its slope and begins to decline, indicating that the operating times are below the mean; in this phase the performance is optimized, the times are reduced, and there is stability. This triphasic pattern mirrors the model of skill acquisition first articulated by Fitts & Posner [13]; and it reflects the typical one described in the literature for other surgical learning curves [14–17]. Although often indicated with different terminology, some studies present in the literature recognize the same three phases identified by us in the learning curve (learning, proficiency and mastery). Other studies identify only two phases in the learning process; nevertheless, the overall pattern of the curves remains the same [14, 18].

Although the SP CUSUM curve does not display the canonical triphasic pattern seen in the MP group, its abbreviated learning trajectory suggests a more efficient adaptation process. The rapid transition from learning to mastery without a proficiency phase indicates that the surgeon was able to bypass much of the intermediate skill refinement typically required in the MP approach. This efficiency likely stems from prior robot-assisted experience and the ergonomic and technological advantages of the SP system. Moreover, this curve features a steady-state phase that is not present in the MP curve. This final segment can be interpreted as a phase of sustained performance or plateau, where the surgeon operates with consistent efficiency and minimal variability. Regarding the leaning rate the near-flat SP slope (− 0.009 min/case) indicates little absolute operative time reduction because the series began close to its mean after prior MP mastery, yet the CUSUM peak occurs early because most SP cases quickly fell below that mean.

The strengths of this study include the fact that, to date, the literature is devoid of analyses and comparisons between the learning curve of the Da Vinci SP with that of the Da Vinci multi-port system, not only in the field of gynecology but in surgical practice more broadly. Another strength of the study is the strong similarity between the patient populations being compared and the fact that all procedures were performed by the same surgeon, ensuring greater consistency and reliability of the results. The study has different limitations. The cohorts were separated by more than a decade, introducing temporal bias (evolving perioperative workflows, institutional experience, and ancillary technologies) that we did not adjust for. In addition, the operating surgeon had performed > 1,500 MP procedures and had prior single-site robotic and single-incision laparoscopic experience before adopting SP [19]; this surgeon bias likely compresses the observed SP learning curve via positive skill transfer. Consequently, our SP curve should be interpreted as the trajectory of platform adoption by an MP-proficient surgeon, not as a generalizable estimate for novice users. These findings are most applicable to high-volume surgeons transitioning from MP to SP. For less-experienced surgeons or training settings, the number of SP cases required to reach proficiency/mastery is likely higher and the trajectory more clearly triphasic. We therefore discourage direct extrapolation of our “early mastery” signal to novice user. To quantify skill transfer definitively, future studies should adopt a contemporaneous, multi-surgeon design. Furthermore, future research on total hysterectomy should also address the learning curve associated with vNOTES and robotic vNOTES procedures performed with the da Vinci SP system, which represent emerging minimally invasive approaches with distinct technical and ergonomic profiles [20, 21].

## Conclusion

In a high-experience setting, the surgical experience gained with the Da Vinci multi-port system appears to be largely transferable to the Da Vinci SP platform. Future studies involving multiple surgeons with varying levels of experience are needed to validate these findings and provide a more comprehensive understanding of the learning dynamics with the SP system.

## Supplementary Information

Below is the link to the electronic supplementary material.

Supplementary Material 1

Supplementary Material 2

## Data Availability

The datasets generated and/or analyzed during the current study are available from the corresponding author on reasonable request.
