# Supplementary material for: Evaluating the learning curve in robot-assisted laparoscopic total hysterectomy: single-port versus multi-port Da Vinci platforms
Source: J Robot Surg. 2025 Nov 1;19(1):733. doi: 10.1007/s11701-025-02928-8 (PMC12579705; doi:10.1007/s11701-025-02928-8)
Supplement: Supplementary file 1 — Supplementary Material 1 [file 11701_2025_2928_MOESM1_ESM.pdf]

REGIONE LAZIO

SISTEMA SANITARIO REGIONALE

TERRITORIAL ETHICS COMMITTEE LAZIO AREA 5

(established by regional determination dated 10/02/2023, n. G01659)

Dr. Enrico Vizza
Department of Gynecologic Oncology - IRE

c.c. **Scientific Direction IRE**

SUBJECT: Territorial Ethics Committee Lazio Area 5 – Extract from Minutes No. 08 dated April 8, 2025 Acknowledgment of Notification for the Robotic Surgery Study LPS versus LESS Experimentation Register No. 322/IRE/25

On April 8, 2025, the Territorial Ethics Committee Lazio Area 5 convened in a hybrid format (both remotely via Microsoft Teams and in person at the meeting room of the Corporate Health Directorate of the I.F.O.).

The Committee, established by Regional Determination No. G01659 dated February 10, 2023, entitled "Reorganization of Territorial Ethics Committees (CET) operating in the Lazio Region pursuant to Legislative Decree No. 3/2018," and based at the I.F.O., comprises members appointed by Determination of the Lazio Region, Directorate of Health and Social Health Integration, No. G07870 dated June 6, 2023, as subsequently supplemented by Determination No. G01598 dated February 10, 2025.

The Istituti Fisioterapici Ospitalieri formally acknowledged the appointments of the members of the Territorial Ethics Committee Lazio Area 5 through Measures No. 565 of June 23, 2023, and No. 283 of March 24, 2025.

Pursuant to Convocation Note Protocol No. 5306 dated April 2, 2025, the following members participated in the meeting:

	Present on site	Present remotely	Absent
**Carlo Tomino** Pharmacologist (President)	x		
**Diana Giannarelli** Biostatistician (Vicepresident)		x	
**Alessia Amore** Bioethics expert		x	
**Pier Luigi Bartoletti** General territorial medicine			
**Alfonso Bellia** Internist Clinician		x	
**Emilio Bria** Medical oncologist		x	
**Raffaele Cozza** Pediatrician onco-hematologist		x	
**Rosa D'Arca** Expert in legal and insurance matters		x	
**Andrea Di Mattia** Medical Device Expert		x	
**Giampiero Forte** Hospital Pharmacist			a.
**Elisabetta Iannelli** Patients' Association Representative		x	
**Maria Rita Lupattelli** Nutrition Expert		x	
**Enrico Marinelli** Medical examiner		x	
**Giovanni Pellacani** Clinical Expert in Dermatology		x	
**Fabrizio Petrone** Health Professions Representative		x	
**Federica Sangiuolo** Expert in Genetics		x	
**Fabrizio Vecchio** Clinical Engineer		x	
**Antonello Vidiri** Expert Clinician new procedures		x	
**Agata Cecilia Amato Mangiameli** Expert in legal matters	x		

REGIONE LAZIO

SISTEMA SANITARIO REGIONALE

**Giuseppe Bruno**	Expert in Neurology		x	
**Alessandro Lambiase**	Esperto in Oculistica		x	
**Giovanni Casella**	Esperto in Chirurgia		x	

The functions of recording secretary are performed by Anna D'Ambrosio, an officer of the I.F.O. and coordinator of the Secretariat of the Territorial Ethics Committee Lazio Area 5.

. . . o m i s s i s . . .

Experimentation Register No. 322/IRE/25 Notification of the Robotic Surgery Study LPS versus LESS "SINGLE-PORT VERSUS MULTIPORT ROBOTIC SURGERY IN PATIENTS WITH GYNECOLOGICAL NEOPLASMS."
PI IFO: Prof. Enrico Vizza - Department of Gynecologic Oncology - IRE
SPONSOR: IFO (no-profit study)
Single-center retrospective study (surgical procedure)

Attachments:

- ❑ **Notification letter CET-DSci dated**
- ❑ **Protocol (version No. 1 – 03.07.2025)**
- ❑ **Protocol signature page**
- ❑ **Information and data processing consent form (version No. 1 – 03.07.2025)**
- ❑ **Declaration of absence of consent**
- ❑ **Non-profit study declaration**
- ❑ **Observational study declaration**
- ❑ **Privacy checklist/DPIA draft**
- ❑ **CV of the Principal Investigator (PI)**

. . . o m i s s i s . . .

The Territorial Ethics Committee Lazio Area 5 acknowledges it.

The Chair of the Territorial Ethics Committee
Lazio Area 5
Prof. Carlo Tomino
