# Supplementary material for: Evaluating the learning curve in robot-assisted laparoscopic total hysterectomy: single-port versus multi-port Da Vinci platforms
Source: J Robot Surg. 2025 Nov 1;19(1):733. doi: 10.1007/s11701-025-02928-8 (PMC12579705; doi:10.1007/s11701-025-02928-8)
Supplement: Supplementary file 2 — Supplementary Material 2 [file 11701_2025_2928_MOESM2_ESM.pdf]

ANALYSIS REPORT
studium

Learning curve final version_SU

13% Suspicious texts

8% Similarities
0% similarities between quotation marks
1% among the sources mentioned
5% Texts potentially generated by AI

Document name: Learning curve final version_SU.docx
Document ID: 0bc7e09344201466e9e337825bf7a826077ebb5a
Original document size: 7.94 MB

Submitter: Riccardo Vizza
Submission date: 8/20/2025
Upload type: interface
analysis end date: 8/20/2025

Number of words: 3,287
Number of characters: 22,088

Location of similarities in the document:

Sources of similarities

Main sources detected

No.	Description	Similarities
1	**pmc.ncbi.nlm.nih.gov** | Lock<br>https://pmc.ncbi.nlm.nih.gov/articles/PMC11032265/	
2	**link.springer.com** | Extraperitoneal Robot-Assisted Radical Prostatectomy | SpringerLink<br>https://link.springer.com/chapter/10.1007/978-3-031-00363-9_26	
3	**pmc.ncbi.nlm.nih.gov**<br>https://pmc.ncbi.nlm.nih.gov/articles/PMC10520215/pdf/464_2023_Article_10252.pdf<br>7 similar sources	

Sources with incidental similarities

No.	Description	Similarities
1	**link.springer.com** | The learning curve in endoscopic transsphenoidal skull-base surgery: a systematic review<br>https://link.springer.com/content/pdf/10.1186/s12893-024-02418-y.pdf	
2	**doi.org**<br>https://doi.org/10.1007/s00464-023-10345-x	
3	**pmc.ncbi.nlm.nih.gov** | Lock<br>https://pmc.ncbi.nlm.nih.gov/articles/PMC6996634/	
4	**doi.org** | Fifty Years at the Forefront of Ethical Guidance: The World Medical Association Declaration of Helsinki<br>https://doi.org/10.14423/smj.0000000000000127	
5	**doi.org**<br>https://doi.org/10.1007/s11701-024-02202-3	

Referenced sources (without similarities detected)

These sources were cited in the paper without finding any similarities.

1 https://doi.org/10.1002/rcs.2453
2 https://doi.org/10.1089/end.2022.0600
3 https://doi.org/10.1080/13645706.2025.2538764
4 https://doi.org/10.1016/j.ejso.2025.110269
5 https://doi.org/10.1111/bju.12197

Points of interest

Evaluating the Learning Curve in Robot-Assisted Laparoscopic Total Hysterectomy: Single-Port versus Multi-port Da Vinci Platforms

Riccardo Vizza1, Simone Garzon1, Giacomo Corrado2, Valentina Bruno3, Ermelinda Baiocco3, Stefano Uccella1, Enrico Vizza3.

1 Unit of Obstetrics and Gynecology, Department of Surgery, Dentistry, Pediatrics, and Gynecology, AOUI Verona, University of Verona, Verona, Italy.
2 UOC Ginecologia Oncologica, Dipartimento di Scienze per la salute della Donna e

1 **pubmed.ncbi.nlm.nih.gov** | Uterine transposition versus uterine ventrofixation before radiotherapy as a fertility sparing option in young women with pelvic malignancies:...
https://pubmed.ncbi.nlm.nih.gov/37992415/

del Bambino e di Sanità Pubblica, Fondazione Policlinico Universitario A. Gemelli, IRCCS, Rome, Italy.

3 Gynecologic Oncologic

2 **hdl.handle.net** | Single cell multi-omic innate immune cell analysis reveals a SARS-CoV-2 specific monocyte phenotype
https://hdl.handle.net/11573/1726529

Unit, Department of Experimental Clinical Oncology, IRCCS Regina Elena National Cancer Institute, Rome, Italy

Author contributions: study conception and design: GC, RV, EV. Literature review: SG, RV. Acquisition of data: EV, GC, EB. Analysis and interpretation of data: SG, RV. Drafting of the manuscript: RV, SG, VB. Critical revision and final approval of the manuscript: EV, SU. All agree to be held accountable for all aspects of the work.
Funding Sources:

3 **link.springer.com**
https://link.springer.com/article/10.1007/s13304-025-02282-2

the authors declare that no funds, grants, or other support were received during the preparation of this manuscript.

Competing Interests: the authors have no relevant financial or non-financial interests to disclose.
Ethical approval: this study was performed in line with the principles of the Declaration of Helsinki. Approval was granted by the Ethics Committee of Lazio Area 5 (RS: 322/IRE/25).
Consent to participate:

4 **doi.org** | Early results of a safety and feasibility clinical trial of a novel single-port flexible robot for transoral robotic surgery
https://doi.org/10.1007/s00405-017-4729-y

informed consent was obtained from all individual participants included in the study.

Consent for publication: not applicable (the manuscript does not contain individual person's data in any form).
Data availability: the datasets generated and/or

5 **trialsjournal.biomedcentral.com** | Who says "no" to participating in stroke clinical trials and why: an observational study from the Vancouver Stroke Program | Trials | Fu...
https://trialsjournal.biomedcentral.com/articles/10.1186/s13063-019-3434-0

analyzed

6 **link.springer.com** | The learning curve in endoscopic transsphenoidal skull-base surgery: a systematic review
https://link.springer.com/content/pdf/10.1186/s12893-024-02418-y.pdf

during the current study are available from the corresponding author on reasonable request.

Corresponding author:
Riccardo Vizza MD,
Unit of Obstetrics and Gynecology, Department of Surgery, Dentistry, Pediatrics, and Gynecology, AOUI Verona, University of Verona.
Piazzale A. Stefani 1, 37126 - Verona, Italy.
riccardo.vizza@studenti.univr.it

Abstract
The aim of this study is to assess and compare the learning curves of the Da Vinci S Multi-port (MP) and Da Vinci Single-Port (SP) platforms for total hysterectomy. This is a retrospective comparative study on patients undergoing robot-assisted total hysterectomy (benign or early endometrial cancer indication) with the MP and SP systems. All operations were performed by the same surgeon, and cases performed with the SP system were performed after the MP system. Operating time was used as the main indicator of the learning curve, analyzed by the CUSUM method. Data were processed with R software, and differences between groups were evaluated with t-test and Chi-squared test (significance $p < 0.05$). A total of 147 patients were analyzed (73 MP, 74 SP). Mean operating times were similar between the two groups (117 min MP vs 114 min SP). Hemoglobin drop was significantly lower in the SP group (−1.18 g/dL vs −2.07 g/dL, $p < 0.05$). CUSUM analysis showed that the learning curve of the MP system reaches the mastery phase after 50 cases, while 13 cases are sufficient for the SP system when initiated after having mastered the MP system. The learning rate was -0.3 min/case for MP and -0.009 min/case for SP. In conclusion much of the experience gained with the Da Vinci Multiport System is transferable to the SP platform, allowing for a shorter learning curve and rapid achievement of surgical mastery with similar learning phases.

Keywords: Learning Curve; Robot-Assisted Hysterectomy, Da Vinci Single-Port, Da Vinci Multi-port.
Introduction
The Da Vinci SP© is a specialized variant of the Da Vinci surgical system tailored for single-port surgery. It utilizes a single robotic arm that passes through one small skin incision. This is made possible by its design, which includes a single flexible camera and three multi-articulated robotic instruments. All these instruments employ two points of articulation within the body, allowing for better angulation toward the surgical field.
The Da Vinci Single Port by Intuitive Surgical, Inc. (Sunnyvale, CA, USA) received approval for gynecologic surgery in South Korea in 2019 and in Japan in 2022. In the United States, while SP was approved in 2019 for select otolaryngology and urology procedures, the approval for gynecologic surgery is still pending. In the EU, the Da Vinci SP has been approved for gynecological procedures in 2024.
Since 2019, the SP system has been demonstrated feasible for several major robot-assisted operations. SP surgery technique was introduced into clinical practice to perform cholecystectomy, colorectal surgery, urological and gynecological surgery, with encouraging preliminary results[1–4].
Given that the SP system was introduced after widespread adoption of the MP platform, it is crucial to understand whether the competencies gained with MP robot-assisted surgery are transferable to SP procedures. This has important implications for surgical training, patient safety, and the efficient integration of new technology into clinical practice. In this context, analyzing the learning curve (LC) becomes particularly relevant, as it represents the relationship between a surgeon's experience and patient outcomes, and it is crucial to estimate the number of patients at risk of suboptimal outcomes during the learning process[5]. This study aims to define the learning curve for the SP platform and the MP platform, with particular

attention to how prior MP experience influences SP performance.

Methods

We conducted a retrospective study to compare the learning curve for the procedure of total hysterectomy performed using the Da Vinci SP© (Intuitive Surgical, Inc., Sunnyvale, CA, USA) system versus the Da Vinci S Multi-port© (Intuitive Surgical, Inc., Sunnyvale, CA, USA) system. The study was approved by the Ethics Committee of the Regina Elena National Cancer Institute of Rome, Italy (RS: 322/IRE/25). Informed consent to surgical intervention, including consent for the retrospective use of clinical data, was obtained from all the

7 www.ncbi.nlm.nih.gov | Single-access laparoscopic approach in the surgical treatment of endometrial cancer: A single-institution experience and review of literature - PMC
https://www.ncbi.nlm.nih.gov/pmc/articles/PMC5022519/

patients in accordance with local and international legislation (Declaration of Helsinki)[6].

We retrospectively reviewed the prospectively maintained operative room database and identified all consecutive total hysterectomy procedures performed with robotic platform by the same single surgeon (EV). We selected the first cases performed by the surgeon with MP and SP system. The involved surgeon had already extensive background in laparoscopic surgery and advanced gynecological oncological procedures before the beginning of the study. The study period was between July 2010 and September 2011 for the MP system and between June 2024 and April 2025 for the SP system. We chose the time interval 2010-2011 for the MP cases, because MP was introduced in July 2010 at our Institution: in this way, we obtained that the involved surgeon did not have previous experience with a robotic MP system.

We identified and included all consecutive robotic-assisted type A and type B1 hysterectomies[7] performed for benign gynecological condition or clinical stage I endometrial cancer. Variables of interest, such as patient demographics, surgical indications, operative times, and estimated blood loss, were retrieved from a prospectively maintained institutional database or collected from medical records by trained physicians.

For the SP group, a single 2.5 cm incision was made at the lower rim of the umbilicus, extending down to the level of the fascia, which was then opened along the longitudinal axis of the body. The leading edge of the folded port was inserted into the incision with a downward motion while countertraction was provided using retractors. A small Intuitive access port was placed into the incision, and pneumoperitoneum was established by insufflating the abdomen to a pressure of 12 mm Hg.

For the MP group, we used the Da Vinci S-system with three robotic arms. After establishing pneumoperitoneum, three 8-mm trocars specific to the Da Vinci robotic system were inserted, along with a 10-mm assistant trocar.

Statistical analysis

Patient demographics and perioperative variables were summarized using standard descriptive statistics, as appropriate, overall and stratified based on the surgical platform: the single-port group (SP) and the Da Vinci S multi-port group (MP). Comparisons between the two groups were performed for continuous variables using the Student's t-test, while differences in nominal categorical variables were assessed with the Chi-squared test.

The cumulative sum (CUSUM) methodology was used for the learning curve analysis, and the primary endpoint was the operative time as best surrogate of learning and proficiency in using the robotic-surgical platform. All surgical cases were sorted by surgical dates. Data for each patient in the series were plotted on a chart from left to right on the x-axis, while the y-axis represents the CUSUM value of operative time. The CUSUM of the operative time was defined as follows: $S_0=0$; $S_i = S_{i-1} + (OT_i -)$. Where S_i is the CUSUM value for case i, S_{i-1} is the CUSUM value for the previous case. OT_i is the observed operative time for case i, and is the mean operative time. The initial CUSUM value is 0 so $S_0=0$. Additionally, for both groups we plotted the raw operative times in chronological order, with a trend line representing the learning rate, modeled through linear regression.

Statistical significance was considered with a p-value < 0.05. All statistical analyses were performed with R Statistical Software (v4.1.2; R Core Team 2024).

Results

Demographic and Clinical Characteristics

A total of 73 consecutive patients who underwent Da Vinci S multi-port (MP) hysterectomy between July 2010 and September 2011, and 74 consecutive patients who underwent Da Vinci SP single-port (SP) hysterectomy between June 2024 and April 2025 were identified and included in the analysis. Demographic and clinical characteristics are summarized in Table 1.

The two groups did not differ in terms of surgical indications. In the MP group, 56 patients had endometrial cancer and 17 had benign gynecological conditions (including endometrial hyperplasia and uterine fibromatosis). In the SP group, 63 patients had endometrial cancer and 11 had benign conditions. The two groups differed significantly in terms of age (mean 58 years in MP vs. 63 years in SP, $p<0.05$), while they were comparable in terms of body mass index (mean BMI 31 kg/m² in MP vs. 29 kg/m² in SP). Regarding operative time no statistically significant difference was observed ($p > 0.05$), with a mean duration of 117 minutes in the MP group and 114 minutes in the SP group. In contrast, the mean postoperative hemoglobin drop was significantly lower in the SP group (1.18 g/dL) compared to the MP group (2.07 g/dL), with this difference reaching statistical significance ($p < 0.05$).

Characteristics Da Vinci MP group (n=73) Da Vinci SP group (n=74) p
Mean Age (years) 58 (SD: 10.9) 63 (SD: 12.2) 0.011
Mean BMI (kg/m²) 31 (SD: 8.1) 29 (SD: 7.3) 0.055
Diagnosis EC n=56, Benign cases n=17 EC n=63, Benign cases n=11 0.193
Mean operative Time (minutes) 117 (SD: 42.7) 114 (SD: 34.2) 0.658
Mean Hb drop (g/dL) 2.07 (SD: 1.1) 1.18 (SD: 0.91) 0.0000327

Table 1: Demographic and clinical characteristics

Learning Curve Analysis

All procedures were performed by the same surgeon; the initial multi-port robot-assisted procedures were performed by the surgeon at a time when he had no prior experience in robot-assisted surgery. In contrast, the first SP cases were carried out after the surgeon had already gained substantial experience with multi-port robot-assisted surgery, although he had no prior clinical experience specifically with the SP platform.

A marked reduction in operative time was observed with increasing experience in the MP group, showing a learning rate of approximately –0.3 minutes per case, indicating a steeper learning curve (Fig. 1A). Conversely, the SP group demonstrates a much more gradual decline in operative time, with a learning rate of –0.009 minutes per case, reflecting a nearly flat learning curve (Fig. 1B). The CUSUM analysis of operative times in the MP group (Fig. 2A) identifies three distinct phases: A learning phase, characterized by a steep upward slope of the curve, corresponding to longer-than-average operative times. A proficiency phase, where the curve continues to rise but with a reduced slope.

A mastery phase, marked by a downward slope, indicating operative times below the average.

In the MP group, the transition to the mastery phase occurred after 50 cases. In contrast, the CUSUM curve of the SP group (Fig. 2B) reveals a different pattern. The learning phase is shorter, with a rapid transition directly into the mastery phase without a clearly distinguishable proficiency phase. This mastery phase, marked by a consistent downward trend in the CUSUM curve, begins around case 13 and continues until case 25.

Beyond this point, the curve flattens, indicating stabilization of operative performance and times oscillating closely around the mean. R2 for MP and SP CUSUM curves is respectively 0.897 and 0.862.

Fig. 1: (A) Operating time as a function of the number of interventions in MP group (B) Operating time as a function of the number of interventions in SP group

Fig. 2: (A) CUSUM Analysis of Operative Time for MP group (B) CUSUM Analysis of Operative Time for SP group

Discussion

The comparative analysis of the learning curves showed that, although there was no statistically significant difference in mean operative times between the da Vinci S Multi-port and the da Vinci Single-Port platforms, the progression of surgical proficiency differed markedly. Specifically, the two platforms exhibited distinct learning curve patterns, indicating differences in the rate and trajectory of skill acquisition.

The CUSUM learning curves showed that the SP system reached the mastery phase after 13 procedures, whereas the multi-port MP system required 50 (Fig. 2B vs Fig. 2A). This compressed trajectory is consistent with the principle of positive skill transfer: once a surgeon has internalized the psychomotor and cognitive workflows of console-based robot-assisted surgery, additional platforms that share similar hand-controller geometry, instrument kinematics and visual feedback impose a far smaller cognitive load. Consequently, only the platform-specific nuances (e.g. adaptation of operative angle and working distance, preventive hemostasis due to smaller instruments) must be learned, while core skills, camera control, three-dimensional depth perception, management of the controls and wristed articulation are already automatized. Comparable accelerations have been reported when surgeons move from MP to SP systems in radical prostatectomy [8, 9] .

The MP curve displays the canonical three phases described in surgical education theory. In the initial learning phase, the curve has a steep upward slope, indicating that the operator is taking longer than the mean operative time. As declarative knowledge is converted into automatized motor patterns, the curve enters the proficiency phase; where the curve begins to flatten but still has an upward slope. Finally, the mastery phase is reached when the curve reverses its slope and begins to decline, indicating that the operating times are below the mean; in this phase the performance is optimized, the times are reduced, and there is stability. This triphasic pattern mirrors the model of skill acquisition first articulated by Fitts & Posner[10]; and it reflects the typical one described in the literature for other surgical learning curves[11–14]. Although often indicated with different terminology, some studies present in the literature recognize the same three phases identified by us in the learning curve (learning, proficiency and mastery). Other studies identify only two phases in the learning process; nevertheless, the overall pattern of the curves remains the same[11, 15].

Although the SP CUSUM curve does not display the canonical triphasic pattern seen in the MP group, its abbreviated learning trajectory suggests a more efficient adaptation process. The rapid transition from learning to mastery without a proficiency phase indicates that the surgeon was able to bypass much of the intermediate skill refinement typically required in the MP approach. This efficiency likely stems from prior robot-assisted experience and the ergonomic and technological advantages of the SP system.

Moreover, this curve features a steady-state phase that is not present in the MP curve. This final segment can be interpreted as a phase of sustained performance or plateau, where the surgeon operates with consistent efficiency and minimal variability,

The strengths of this study include the fact that, to date, the literature is devoid of analyses and comparisons between the learning curve of the Da Vinci SP with that of the Da Vinci multi-port system, not only in the field of gynecology but in surgical practice more broadly. Another strength of the study is the strong similarity between the patient populations being compared and the fact that all procedures were performed by the same surgeon, ensuring greater consistency and reliability of the results. The main limitation of the study is that all procedures were performed by a surgeon with many years of experience in laparoscopic surgery. Moreover, before starting to use the Da Vinci SP system, the surgeon had already performed over 1,500 procedures with the Da Vinci multi-port system and had prior experience with both single-site robot-assisted surgery and single-incision laparoscopic surgery. This extensive prior experience may have influenced the observed learning curve for the Da Vinci SP. This limits the generalizability of the results to less experienced surgeons or training contexts.

Conclusion

The surgical experience gained with the Da Vinci multi-port system appears to be largely transferable to the Da Vinci SP platform. Future studies involving multiple surgeons with varying levels of experience are needed to validate these findings and provide a more comprehensive understanding of the learning dynamics with the SP system.

Acknowledgments

The authors wish to thank the nursing staff of the Gynecologic Oncology Unit of the Regina Elena National Cancer Institute for their assistance during the surgery.

Bibliography

1. Bianco FM,

8 **pubmed.ncbi.nlm.nih.gov** | Robotic single-port surgery: Preliminary experience in general surgery - PubMed
https://pubmed.ncbi.nlm.nih.gov/35962708/

Dreifuss NH, Chang B, Schlottmann F, Cubisino A, Mangano A,
Pavelko Y, Masrur MA, Giulianotti PC (2022) Robotic single-port surgery: Preliminary experience in general surgery. Int J Med Robot 18:e2453. https://doi.org/10.1002/rcs.2453

2. Nguyen TT, Basilius J, Ali SN, Dobbs RW, Lee DI (2023) Single-Port Robotic Applications in Urology. J Endourol 37:688–699. https://doi.org/10.1089/end.2022.0600

3. Vizza R, Corrado G, Mancini E, Baiocco E, Russo M, Vincenzoni C, Bruno V, Falconer H, Vizza E (2025) Feasibility, safety, and efficacy of robotic single-port hysterectomy (R-SPH) using the da Vinci SP system in low-risk endometrial cancer: a pilot study. Minim Invasive Ther Allied Technol 1–8. https://doi.org/10.1080/13645706.2025.2538764

4. Vizza E, Giannini A, Bruno V, Baiocco E, Mancini E, Vizza R, Uccella S, Raspagliesi F, Bogani G (2025) Robotic-assisted single-port and multi-port surgical staging in early-stage endometrial cancer: a propensity matched comparison. Eur J Surg Oncol 110269. https://doi.org/10.1016/j.ejso.2025.110269

9 **pmc.ncbi.nlm.nih.gov** | Lock
https://pmc.ncbi.nlm.nih.gov/articles/PMC6996634/

5.

10 **link.springer.com** | The learning curve in endoscopic transsphenoidal skull-base surgery: a systematic review
https://link.springer.com/content/pdf/10.1186/s12893-024-02418-y.pdf

Khan N, Abboudi H, Khan MS, Dasgupta P, Ahmed K (2014) Measuring the surgical 'learning curve': methods, variables and competency. BJU Int
113:504–508. https://doi.org/10.1111/bju.12197

6. (2013)

11 **doi.org** | Fifty Years at the Forefront of Ethical Guidance: The World Medical Association Declaration of Helsinki
https://doi.org/10.14423/smj.0000000000000127

World Medical Association Declaration of Helsinki: Ethical Principles for Medical Research Involving Human Subjects. JAMA
310:2191. https://doi.org/10.1001/jama.2013.281053

7. Querleu D, Morrow CP (2008) Classification of radical hysterectomy. Lancet Oncol 9:297–303. https://doi.org/10.1016/S1470-2045(08)70074-3

8.

12 **link.springer.com** | Extraperitoneal Robot-Assisted Radical Prostatectomy | SpringerLink
https://link.springer.com/chapter/10.1007/978-3-031-00363-9_26

Covas Moschovas M, Bhat S, Onol F, Rogers T, Patel V (2021) Early outcomes of single-port robot-assisted radical prostatectomy: lessons learned from the learning-curve experience. BJU Int 127:114–121. https://doi.org/10.1111/bju.15158

9.

13 **pmc.ncbi.nlm.nih.gov** | Lock
https://pmc.ncbi.nlm.nih.gov/articles/PMC11032265/

Ramos-Carpinteyro R, Ferguson EL, Chavali JS, Geskin A, Soputro N, Kaouk J (2023) Single-port Transvesical Robot-assisted Radical Prostatectomy: The Surgical Learning Curve of the First 100 Cases. Urology
178:76–82. https://doi.org/10.1016/j.urology.2023.05.027

10. Fitts PM, Posner MI (1967) Human performance. Brooks/Cole Publishing Company

11. Lombardi PM, Mazzola M, Veronesi V, Granieri S, Cioffi SPB, Baia M, Del Prete L, Bernasconi DP, Danelli P, Ferrari G (2023)

14 doi.org
https://doi.org/10.1007/s00464-023-10345-x

Learning curve of laparoscopic cholecystectomy: a risk-adjusted cumulative summation (RA-CUSUM) analysis of six general surgery residents. Surg Endosc 37:8133–8143. https://doi.org/10.1007/s00464-023-10345-x
12. Maayan O, Pajak A, Shahi P, Asada T, Subramanian T, Araghi K, Singh N, Korsun MK, Singh S, Tuma OC, Sheha ED, Dowdell JE, Qureshi SA, Iyer S (2023) Percutaneous Transforaminal Endoscopic Discectomy Learning Curve: A CuSum Analysis. Spine 48:1508–1516. https://doi.org/10.1097/BRS.0000000000004730
13. Gil PJ, Ruiz-Manzanera JJ, Ruiz de Angulo D, Munitiz V, Ferreras D, López V, Conesa A, Ortiz Á, Martínez de Haro LF, Ramírez P (2022)

15 doi.org | Learning Curve for Laparoscopic Sleeve Gastrectomy: a Cumulative Summation (CUSUM) Analysis
https://doi.org/10.1007/s11695-022-06145-2

Learning Curve for Laparoscopic Sleeve Gastrectomy: a Cumulative Summation (CUSUM) Analysis. Obes Surg 32:2598–2604. https://doi.org/10.1007/s11695-022-06145-2
14. Li X, Guo S, Yao K, Ge Z, Li Y, Hu J,

16 doi.org | Learning curve of transanal minimally invasive surgery for rectal neoplasm
https://doi.org/10.3389/fonc.2025.1545589

Xia H (2025) Learning curve of transanal minimally invasive surgery for rectal neoplasm. Front Oncol 15:1545589. https://doi.org/10.3389/fonc.2025.1545589
15. Wu J, Wang Y, Huang Y, Long X, Tang J, Gu D (2025)

17 doi.org
https://doi.org/10.1007/s11701-024-02202-3

Learning curve analysis of extraperitoneal single-site robotic-assisted radical prostatectomy: a CUSUM-based approach. J Robot Surg 19:49. https://doi.org/10.1007/s11701-024-02202-3
